# Current status of PSMA-targeted imaging and therapy

**DOI:** 10.3389/fonc.2023.1230251

**Published:** 2024-01-09

**Authors:** Hui Wang, GuanNan Li, Jie Zhao, Matthias Eiber, Rong Tian

**Affiliations:** ^1^ Department of Nuclear Medicine, West China Hospital, Sichuan University, Chengdu, China; ^2^ Department of Nuclear Medicine, Sanmenxia Central Hospital, Henan, China; ^3^ Department of Nuclear Medicine, Klinikum rechts der Isar, Technical University of Munich, Munich, Germany

**Keywords:** PSMA, PET, prostate cancer, theranostics, radioguided surgery

## Abstract

Currently, the incidence of prostate cancer is increasing, and it has become a great threat to men’s health. The detection, staging, and follow-up of prostate cancer patients are inseparable from morphology or magnetic resonance imaging (MRI). However, these do not fully meet the needs of diagnosis and patient management. In particular, owing to the late diagnosis, metastatic castration-resistant prostate cancer (mCRPC) patients usually have poor survival and few options for further effective treatment. Prostate-specific membrane antigen (PSMA), because of its overexpression on prostate cancer cells, has gained interest due to its application in the imaging and theranostics field. Several PSMA radioligands have been developed for imaging and treating prostate cancer. Many clinical trials have assessed the efficacy and safety profiles of these radionuclide agents and show promise in patients who have exhausted other standard treatment options. To date, several small compounds for targeting PSMA have been developed, and ^68^Ga-PSMA-11 and ^18^F-DCFPyL have been approved by the United States (US) Food and Drug Administration (FDA) for imaging of prostate cancer. ^111^In- or ^99m^Tc-labeled PSMA-ligand can guide surgeons searching for radioactive metastatic lymph nodes, and ^177^Lu- or ^225^Ac-labeled PSMA-ligand can be used for internal radiotherapy. Moreover, some molecules for therapeutic application are undergoing different stages of clinical trials. In this review, we present current perspectives on the use of PSMA-targeted imaging and theranostics in prostate cancer. As PSMA-targeted imaging and therapeutics are becoming the standard of care for prostate cancer patients, we emphasize the importance of integrating nuclear medicine physicians into multidisciplinary oncology teams.

## Introduction

Prostate cancer (PCa) ranks third among the most common cancers diagnosed in men worldwide. With approximately 2.3 million new cases by 2040, PCa is becoming one of the most prevalent neoplasms in men worldwide (1, 2). Thus, accurate diagnosis, staging, and effective personalized treatment methods are of vital importance.

Based on anatomy information, conventional imaging strategies such as computed tomography (CT) and magnetic resonance imaging (MRI) have limitations in lesion detection, especially in metastatic patients, while bone scans do not offer anatomical details. Thus, novel molecular imaging biomarkers are urgently needed. Prostate-specific membrane antigen (PSMA) is a type II transmembrane glycoprotein encoded by folate hydrolase 1 gene (FOLH1) (3–5). It is weakly expressed in healthy prostate tissue but is overexpressed in PSMA-positive PCa cells (3). Furthermore, increasing expression of PSMA expression is observed in androgen deficiency, hormone refractory and metastatic diseases, higher PSA levels, and a higher International Society of Urologic Pathologists (ISUP) grade at diagnosis (6, 7). Primary PCa lesions are PSMA-negative proximately in 5%–10% of cases (8). Moreover, PSMA is not specific to the prostate, and the expression in the neovasculature of other solid malignancies (such as renal cell, bladder transitional cell, and colon cell) was observed (3). Moreover, in numerous normal tissues, for example, endometrial glands, testis, bladder, kidney tubules, pancreas islets, heart, and ganglion cells in the gastrointestinal tract and brain, weak-to-moderate levels of PSMA expression have been detected (9). Although it is reported that certain benign diseases such as lymphadenitis and malignant diseases such as lung adenocarcinoma may also exhibit PSMA uptake (10), its remarkable sensitivity and specificity for PCa continue to establish it as a dedicated imaging modality for patients with PCa. Thus, PSMA-positron emission tomography (PET) is still considered to be a rather sensitive and highly specific tool for PCa diagnosis despite its expression by subsets of various types of other tissues (9, 11, 12).

In the field of PSMA-related therapy, PSMA-ligand imaging can help improve the prognosis. Felix et al. combined PSMA-PET and MRI-guided single-component stereotactic radiotherapy (SBRT) for local recurrence of PCa, and the findings are encouraging (13). Furthermore, several clinic trials such as VISION trial (14), TheraP trial (15), and LuPSMA trial (16) have demonstrated promising results of PSMA-targeted therapy in PCa patients. Moreover, Horn et al. found that PCa patients with recurrence at a single anatomical interval of location experienced significantly longer biochemical recurrence-free survival (bRFS) and treatment-free survival interval through PSMA radioguided surgery (RGS) (17).

This review will provide an overview of the current clinical applications of PSMA-targeted imaging and therapy.

## PSMA-ligand imaging

PSMA-ligand PET/CT or MRI is mainly used in the following clinical indications: primary staging of cancers, biochemical recurrence (BCR), and detecting and monitoring of advanced disease.

### Primary staging

A precise staging, local extent, and extra-prostatic metastasis are crucial in intermediate-risk and high-risk PCa patients for further treatment method decisions. These include radical prostatectomy (RP) with standard nodal dissection or extended pelvic lymph node dissection (ePLND) in PCa, radiotherapeutic treatment, or multimodal therapy. A prospective comparison of primary staging before and after PSMA-ligand PET/CT was conducted. Intermediate- and high-risk PCa patients (*n* = 108) have indicated that PSMA-ligand PET/CT upstaged 6.4% of patients from M0 to M1. Moreover, compared with conventional imaging, 14% of N0M0 patients became N1M0, and 2.8% of M1 patients ended up with M0. A change in management of the disease occurred in 21% of patients (18).

The additional molecular imaging information has increased the detection rate of PSMA-ligand PET/CT. As evidence increases, PSMA-ligand PET/CT is more effective in initial staging than other imaging modalities. Compared with traditional imaging modalities such as bone scans and CT, several studies have shown ^68^Ga-PSMA-11 PET improved detection rates in malignant lesions (19). Several retrospective studies have shown that ^68^Ga-PSMA-11 PET/CT is superior to standard imaging and has high sensitivity and specificity (94% and 99%, respectively) for lesion detection (12, 20). A recent prospective trial, proPSMA, demonstrated that the diagnostic accuracy of PET/CT was 27% greater than conventional imaging (*p* < 0.001) (21).

**Figure 1:**
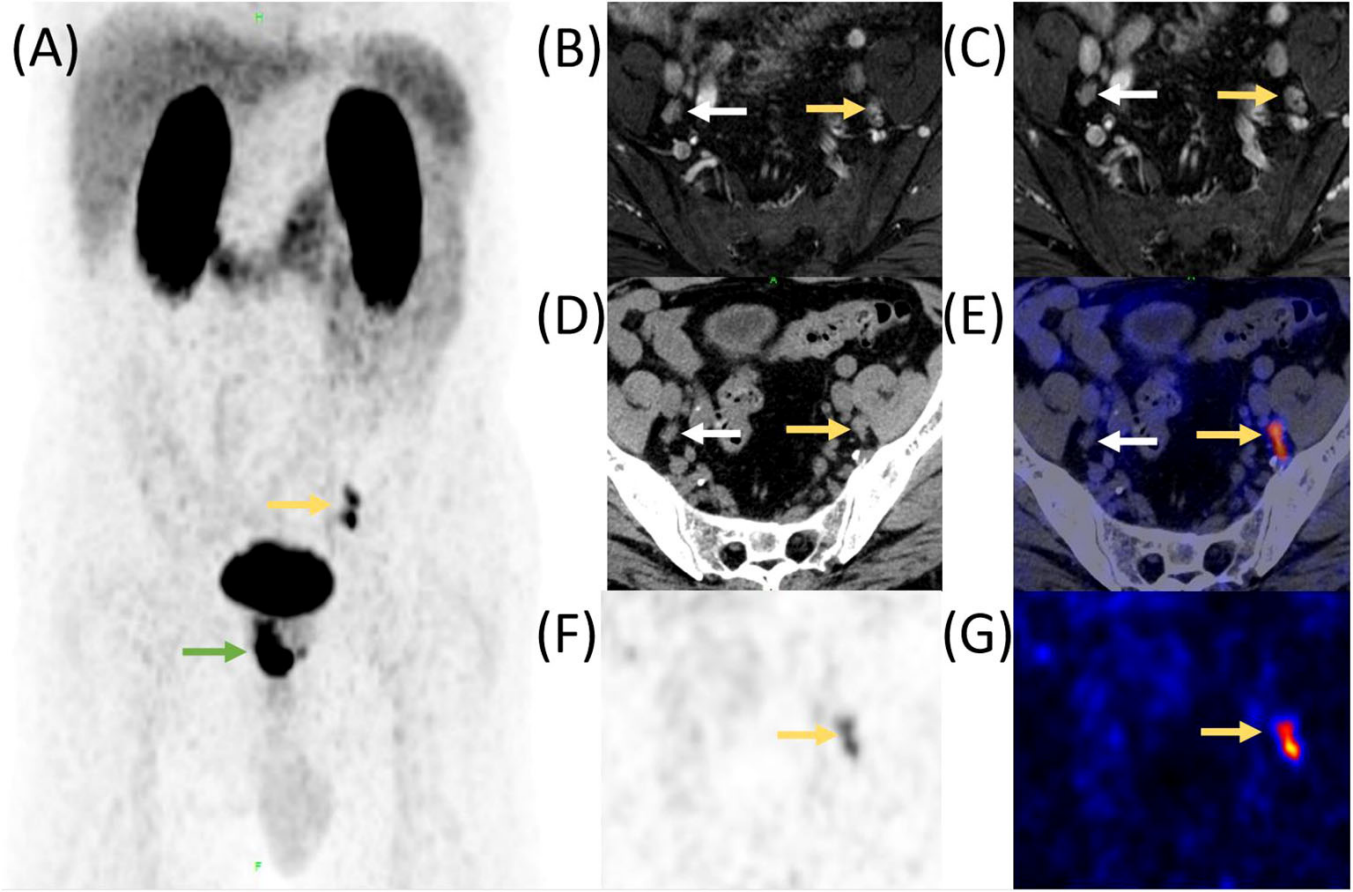
Comparison of enhanced MR, CT, and ^68^Ga-PSMA-11 PET/CT in a PCa patient with lymph node metastasis before RP. A 55-year-old man was diagnosed with prostate cancer;, MR and ^68^Ga-PSMA-11 PET/CT was performed before RP. High ^68^Ga-PSMA-11 uptake was observed in the lower left abdomen and pelvis (**A**, yellow and green arrow). The preoperative MR identified several lymph nodes with a maximum of 5 mm in short diameter, adjacent to the internal iliac vessels bilaterally (**B**, axial enhanced T2-weighted phase 1 MR image; **(C)**, axial enhanced T2-weighted phase 2 MR image, yellow and white arrow). CT also revealed those lymph nodes (**D**, yellow and white arrow), but PET/CT fusion image **(E)**, PET image **(F)**, and **“**hot iron**”** pseudocolor PET image **(G)** demonstrated that only the lymph nodes on left side had high ^68^Ga-PSMA-11 uptake. Postoperative histopathology confirmed that the left lymph nodes were metastasis while the right one was reactive hyperplasia.

The high specificity and high sensitivity of PSMA-ligand PET in lesion detection of primary PCa patients have been proven. A retrospective study of 1,253 patients (high-risk, 47.6%), metastatic disease was detected by ^68^Ga-PSMA-11 PET in 12.1% of the cohort. Nearly half of the detected lymph node metastases were outside the boundaries of an ePLND (**Figure 1**). Skeletal metastases were found in 59 men (4.7%). PCa metastases were identified in 5.2% of intermediate-risk patients, compared with 19.9% of high-risk patients. The study highly demonstrated the potential of using PSMA-PET for primary staging (22). Another study comparing 340 segments from ^68^Ga-PSMA-11 PET and multiparametric MRI with histopathology has revealed that ^68^Ga-PSMA-11 PET/CT significantly outperformed multiparametric MRI and even better results were achieved when both methods were combined (23).

Furthermore, for detection of bony lesions in PCa, PSMA-ligand PET clearly outperformed standard imaging (CT, MRI, and bone scanning) in several studies (24, 25). Janssen et al. have reported a superior sensitivity and specificity of ^68^Ga-PSMA-11 PET/CT compared with ^99m^Tc-3,3-diphospho-1,2-propanodicarboxylic acid (DPD), single-photon emission computed tomography (SPECT). In the region-based analysis, the sensitivity and specificity of ^68^Ga-PSMA-11 PET/CT were 97.7% and 100%, respectively, and those of ^99m^Tc-DPD-SPECT were 69.4% and 98.3%, respectively (*p* < 0.05) (25). A study including 75 patients and 410 bone regions has demonstrated similar results. It has been proven that ^68^Ga-PSMA-11 PET outperformed planar ^99m^Tc bone scintigraphy to detect affected bone regions (sensitivities and specificities for PET vs. ^99m^Tc bone scintigraphy [patient-based analysis]: 98.7%–100% vs. 86.7%–89.3%; 88.2%–100% vs. 60.8%–96.1%; [region-based analysis]: 98.8%–99% vs. 82.4%–86.6%; 98.9%–100% vs. 91.6%–97.9%; *p* < 0.001, respectively) (24). Interestingly, according to a systematic review of 12 studies and 322 patients who underwent ^68^Ga-PSMA-11 PET scanning for primary stage disease, methodology and outcomes differed greatly. The median sensitivity and specificity were 33%–92%, 82%–100% per lesion and 82%–100%, 67%–99% per patient, respectively.

Several studies indicated that in most tumor diseases, the diagnostic performance of PET/MRI is similar to or even better than that of PET/CT (26). PET/MRI is widely utilized in PCa patients, primarily for its advantages in combining PET and MRI, providing high-resolution multiparametric imaging (27). PET/MRI is employed to delineate radiation therapy target areas (28) and diagnose BCRs (29), and for the preoperative assessment of high-risk patients (30). This technology has the potential to enhance the management and treatment decision-making for PCa patients, especially in complex cases, but further research is needed to validate its advantages.

Therefore, PSMA-ligand PET/CT or PET/MRI can be used to determine the extent of local tumors, lymph nodes, bone, and organ metastases, and, more importantly, to plan effective treatment and improve prognosis. However, published data on mechanism experiments and heterogeneous uptake of PSMA-ligand are still limited (31). Recent studies have demonstrated this using PSMA immunohistochemistry; nonetheless, PSMA ligands and antibodies have different sizes and binding positions, which may introduce methodological limitations. Further studies are needed before reliable conclusions can be drawn.

### Biochemical recurrence detection

In approximately 30% to 40% of PCa cases, disease will recur after the primary treatment. Elevating levels of prostate-specific antigen (PSA) always indicate the recurrence (11). Because of the implications for future disease management, the location of the lesions must be determined once BCR has been detected. However, localizing lesions is a difficult task. There has been evidence that salvage radiotherapy in patients with BCR after RP is most effective while serum PSA <0.5 ng/mL (32, 33). Radiotherapy timing is crucial to these patients. Monitoring PSA cannot easily provide enough information.

Regarding conventional imaging modalities, CT can detect only 11%–14% of lesions in patients with BCR after RP (34). Compared with bone scans, choline-based PET imaging has higher pooled specificity [0.82, 95% confidence interval (CI) 0.78, 0.85; 0.99, 95% CI 0.93, 1.00, respectively] and reports fewer false-positive lesions (35). Moreover, the PSA level, kinetics, and morphology have significantly affected the sensitivity of choline PET (36–38).

**Figure 2:**
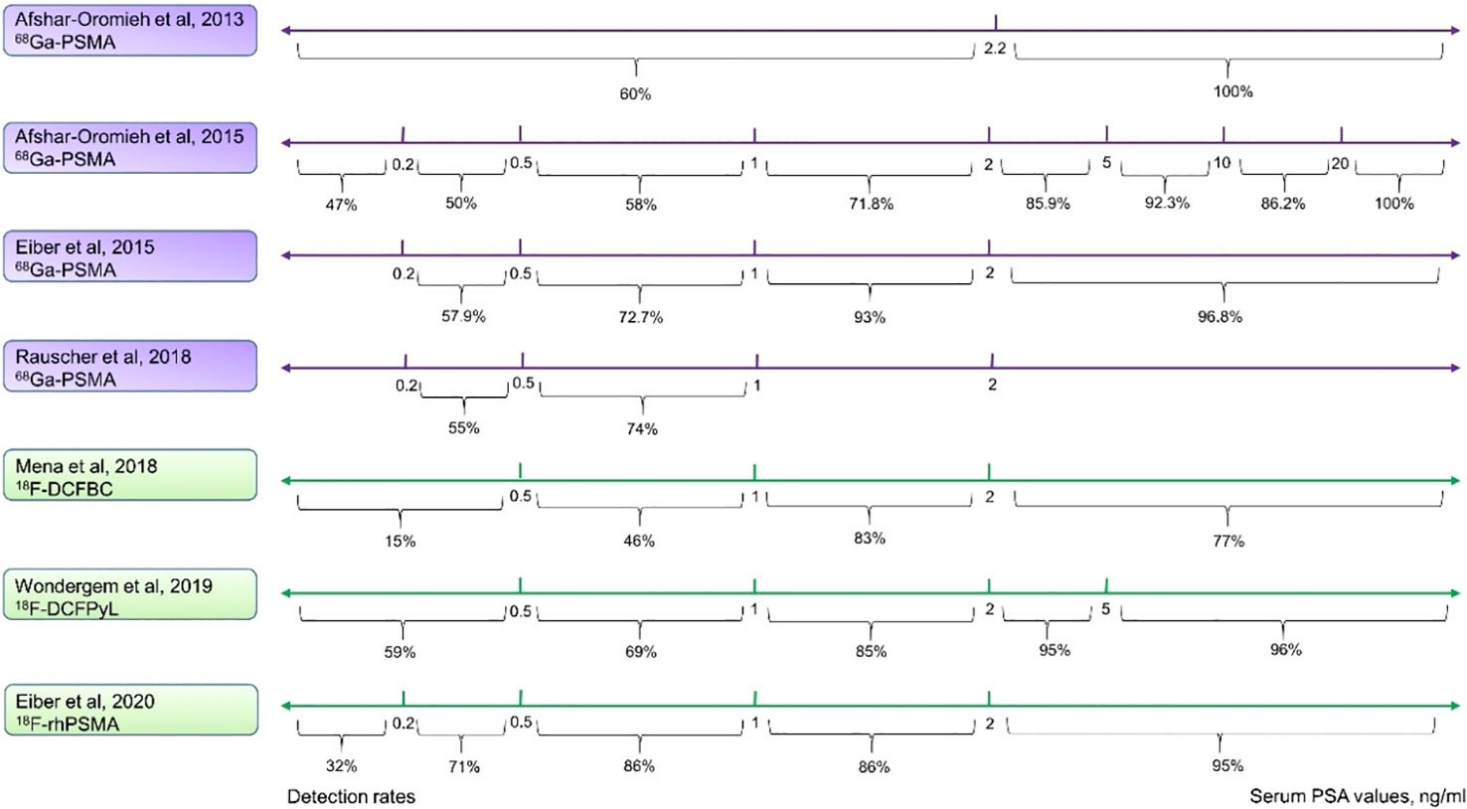
Comparison of detection rates of ^68^Ga and ^18^F labeled PSMA-ligand. Purple lines indicate the ^68^Ga-PSMA-11, and green lines show ^18^F-PSMA-ligands. The ^68^Ga-PSMA-11 PET has a modestly high detection rate even at low serum PSA levels. ^18^F-DCFPyL has a higher detection rate in all the various PSA levels as compared with ^18^F-DCFBC and is comparable with ^68^Ga-PSMA-11. ^18^F-rhPSMA performs the best among these ^18^F-labeled ligands and is similar to ^68^Ga-PSMA-11.

However, PSMA-ligand PET shows a much higher detection rate, especially in low PSA levels. Afshar-Oromieh et al. assessed a large cohort including 319 patients with recurrent PCa who underwent ^68^Ga-PSMA-11 PET (median serum PSA value of 4.59 ng/mL, range: 0.01–41395 ng/mL). They reported detection rates of 47% for serum PSA values ≤ 0.2 ng/mL, rising to 100% for serum PSA values > 20 ng/mL (39). Similar results from other studies have further proven that ^68^Ga-PSMA-11 PET has higher detection rate for BCR with low PSA levels (40, 41). In addition, data from other research groups that studied ^18^F-DCFBC, ^18^F-DCFPyL, and ^18^F-rhPSMA-7 have also indicated rather high detection rates with low serum PSA levels (**Figure 2**) (42–44).

However, these results should be treated with caution because there was no systematic histological confirmation in most of the patients, and follow-up imaging was incomplete in the entire cohort. Current guidelines do not make recommendation for a single radiopharmaceutical. The comparison among different PSMA-targeted tracers are missing; thus, the term “PSMA-ligand” refers to a number of different tracers (45). It is necessary to perform further clinical studies with standardized follow-up protocols and histological validation before comparing outcomes associated with the use of these tracers.

### Advanced disease monitoring

Androgen deprivation therapy (ADT) is the mainstay of treatment for PCa patients with recurrent or advanced disease (46, 47). However, in the first 5 years following ADT, approximately 10%–20% of patients will develop castration-resistant PCa (CRPC) (48). Once the metastatic castration-resistant prostate cancer (mCRPC) is detected, the overall survival (OS) ranges from 2 to 3 years (46, 47).

Monitoring the serum PSA level provides insight into both the disease burden and the biology of the disease; however, some mCRPC patients may have a low PSA level, because the tumor is less dependent on androgen receptor signaling. Moreover, PSA levels can stay low while visceral metastases are developing (49). The relationship between increasing PSA values and disease progression has been confirmed, but decreasing PSA values are not always associated with response to therapy. Using serum PSA alone to monitor disease development or therapy response in mCRPC patients is not entirely reliable (47, 50).

For mCRPC patients, some studies have suggested the choline PET/CT may be used in disease monitoring (51, 52). However, another prospective study has reported that there was no significant correlation between ^11^C-choline PET/CT image findings and chemotherapy treatment response (53). In summary, the application of choline PET/CT appears to be limited to the routine monitoring of mCRPC patients.

It has been demonstrated that new tracers, such as ^68^Ga-PSMA-11 PET/CT (54), can be used for monitoring disease in addition to choline, although these are not yet recommended in the mCRPC patient guidelines (45). Gafita et al. have reported a case of an mCRPC patient who underwent ^177^Lu-PSMA therapy, and the ^68^Ga-PSMA PET has clearly reflected the lesion alteration during the therapy (55). Thus, further studies in mCRPC patients are urgently needed to confirm the potential clinical application of PSMA-ligand in disease monitoring.

## PSMA-targeted radiation therapy

Several therapies have been approved for mCRPC patients; however, the survival benefit in patients is usually less than 6 months (56). Novel therapies are needed for clinical application, and PSMA has emerged as a promising therapeutic molecular target. There are mainly two types of radioisotopes: alpha-emitting and beta-emitting.

### Beta-emitting PSMA-targeted radioligand therapies

^177^Lu-PSMA-617 is the most used beta-therapy molecule. The TheraP phase 2 trial reported that ^177^Lu-PSMA-617 led to a higher PSA response rate (65/98 vs. 37/85) and fewer adverse events (32/98 vs. 45/85) as compared with cabazitaxel (15). Furthermore, the VISION trial has reported that compared with standard care, ^177^Lu-PSMA-617 plus standard care significantly prolonged both imaging-based progression-free survival (median, 8.7 vs. 3.4 months; hazard ratio for progression or death, 0.40; 95% CI, 0.29, 0.57; *p* < 0.001) and OS (median, 15.3 vs. 11.3 months; hazard ratio for death, 0.62; 95% CI, 0.52 to 0.74; *p* < 0.001). Additionally, the quality of life was not affected by the adverse events caused by ^177^Lu-PSMA-617 therapy (14). Another trial using LuPSMA reported that of 30 patients with mCRPC who had progressed after conventional treatment, 17 achieved a PSA decline of 50% or more. Fewer toxic effects and a reduction in pain were observed (16).

The other commonly used PSMA-ligand is ^177^Lu-PSMA-I&T, which also shows promising results (57). In a study of 100 patients where ^177^Lu-PSMA-I&T was used, a good response to treatment and low toxicity were observed (PSA decline ≥ 50%: 38 patients had a median clinical progression-free survival of 4.1 months and median OS was 12.9 months) (58). Several studies (59–61) have shown similar results (summarized in **Table 1**).

**Table 1:** Studies of PSMA-targeted therapy.

Radioligand	Clinical Trial Identifier/Authors	Primary Study Objective
**^177^Lu-PSMA-617**	NCT03392428	^177^Lu-PSMA-617 vs. cabazitaxel
**^177^Lu-PSMA-617**	NCT03511664	^177^Lu-PSMA-617 + standard care vs. standard care alone
**^177^Lu-PSMA-617**	NCT04343885	^177^Lu-PSMA-617 + docetaxel vs. docetaxel in mHSPC
**^177^Lu-PSMA-617**	12615000912583	mCRPC patients who progressed after conventional treatment
**^177^Lu-PSMA-617**	Fendler et al.	Dosimetry, safety, and efficacy evaluation
**^177^Lu-PSMA-617**	Rahbar et al., 2016	Safety and efficacy as 3rd-line therapies in mCRPC
**^177^Lu-PSMA-617**	Rahbar et al., 2018	Survival evaluation of heavily pretreated mCRPC
**^177^Lu-PSMA-I&T**	NCT04886986	Effectiveness of ^225^Ac-J591 and ^177^Lu-PSMA-I&T combination for mCPRC
**^177^Lu-PSMA-I&T**	NCT04297410	^177^Lu-PSMA-I&T Radionuclide Neo-Adjuvant Treatment Feasibility Trial
**^177^Lu-PSMA-I&T**	NCT05204927	^177^Lu-PSMA-I&T vs. Hormone Therapy
**^177^Lu-PSMA-I&T**	Heck et al.	Survival evaluation of heavily pretreated mCRPC
**^177^Lu-J591**	NCT03545165	Combination of ^177^Lu-J591 and ^177^Lu-PSMA-617
**^177^Lu-J591**	NCT00195039	Effectiveness of ^177^Lu-J591 for mCPRC
**^177^Lu-J591**	NCT00859781	Effectiveness of ^177^Lu-J591 in combination with ketoconazole and hydrocortisone against BCR.
**^177^Lu-J591**	NCT00916123	Effectiveness of ^177^Lu-J591 in combination with docetaxel in mCRPC
**^225^Ac-J591**	NCT04506567	Dose-escalation study of fractionated ^225^Ac-J591 for mCPC
**^227^Th-PSMA-TTC**	NCT03724747	Safety and efficacy in mCRPC

BCR, biochemical recurrence; mCRPC, metastatic castration-resistant prostate cancer; mHSPC, metastatic hormone-sensitive prostate cancer; PSMA, prostate-specific membrane antigen.

In addition, ^177^Lu-PSMA-617 has been used in several types of combination therapies. In CRPC patients, the combination of androgen-receptor-axis-targeted therapies (ARATs) may lead to improved tumor control. The DNA damage repair (DDR) pathway has been proven to participate closely during the radioligand therapy (RLT) (62); hence, the combination with PARP inhibitors such as olaparib and rucaparib have been under analysis. Moreover, immune checkpoint inhibitors such as anti-PD-1 or anti-CTLA-4 monoclonal antibodies are an important class of cancer therapies. Owing to the abscopal effect, combination therapy is under evaluation. **Table 2** summarizes representative clinical trials of combination therapies.

**Table 2:** Studies of 177Lu-PSMA-617 combination therapies.

Clinical Trial Identifier	Combination Method
**ENZA-P (NCT04419402)**	Enzalutamide in mCRPC
**PSMAddition (NCT04720157)**	ARATs and ADT in mHSPC
**LuPARP (NCT03874884)**	Olaparib in mCRPC
**PRINCE (NCT03658447)**	Pembrolizumab in mCRPC

ARATs, androgen-receptor-axis-targeted therapies; mCRPC, metastatic castration-resistant prostate cancer; mHSPC, metastatic hormone-sensitive prostate cancer; PSMA, prostate-specific membrane antigen.

There are other notable PSMA-targeting radionuclide therapy agents. ^131^I-MIP-1095 is one of the first agents used in radiopharmaceutical therapy against PCa (63). A study of 34 mCRPC patients who received ^131^I-MIP-1095 found that the first dose of RLT presented with the best therapeutic effect compared with the second and the third doses (64). ^177^Lu-PSMA-R2 is a urea-based PSMA-targeting small-molecule inhibitor (63). It has been shown to present a rapid tumor uptake and elimination through the urinary system in preclinical studies (65). A phase 1/2 clinical trial to evaluate ^177^Lu-PSMA-R2 in mCRPC is currently under way (NCT03490838).

### Alpha-emitting PSMA-targeted radioligand therapies

Utilizing alpha emitters for treatment offers two distinct advantages compared to conventional radioligand therapies. The short distance between cells within human tissue (<0.1 mm) allows the selective destruction of target cancer cells while preserving the surrounding healthy tissue (66). The two most commonly used alpha-emitting radioactive isotopes currently are ^225^Ac and its daughter nuclide ^213^Bi, with half-lives of 9.9 days and 46 min, respectively.

Recent studies have demonstrated the remarkable efficacy of ^225^Ac-PSMA-617 in the treatment of mCRPC, particularly in patients who have experienced disease progression after ^177^Lu-PSMA therapy (67). There is also a clinical case report of 213Bi-PSMA-617, which is both an alpha and beta emitter, that achieved a drop in PSA from 237 μg/L to 43 μg/L in the patient with mCRPC (68). The study by Sathekge et al. (69) involved a relatively small cohort of 21 patients, and confirmed that ^225^Ac-PSMA-617 can lead to a significant reduction of pre-treatment PSA levels by more than 50% in the majority of hormone-sensitive prostate carcinoma (mHSPC) patients.

However, the use of high-activity ^225^Ac/^213^Bi generators for preparing therapeutic doses in the gigabecquerel (GBq) range remains prohibitively expensive, and the global supply of ^225^Ac produced from ^229^Th is severely limited (66). In terms of treatment, due to its highly cytotoxic nature, alpha-emitting isotopes like ^225^Ac may be associated with more serious side effects compared with PSMA-targeted β-radiation, including conditions like xerostomia (70). Furthermore, ^225^Ac produces a substantial number of recoiling daughter nuclei during its decay process, making this radioisotope challenging to control as a targeted alpha therapy agent (71). Nonetheless, this form of treatment offers hope for mCRPC and mHSPC patients and underscores the significant potential that targeted alpha therapy may have in the treatment of other cancers.

### PSMA-targeted immunotherapies

There are several anti-PSMA radioimmunotherapies under evaluation, with a notable example being the J591 antibody. ^177^Lu-J591, ^255^Ac-J591, and ^227^Th-PSMA-TTC are three promising antibodies also under evaluation. The preliminary results using J591 show high efficacy and few side effects (72). The related clinical trials are listed in **Table 1**.

PSMA-targeted bispecific T-cell engager (BiTE) immunotherapy has been developed in recent years. Pasotuxizumab (AMG 212) binds to CD3 on T cells and PSMA on the tumor cells. A phase I clinical trial (NCT 01723475) has shown that a PSA decrease ≥ 50% was observed in 3 out of 16 patients and that BiTE immune therapy could be efficacious in solid tumors (73).

## PSMA radioguided surgery

In patients with PCa, metastatic or recurrent lymph nodes may be in atypical locations and/or small in morphology, hindering accurate identification before and at the time of potential surgery (74).

Owing to the high detection rate for lymph node metastases, PSMA-ligand imaging combined with lymphadenectomy and salvage lymph node resection are increasingly used. PSMA-RGS allows the detection of PSMA-positive PCa cells and metastatic lymph nodes during the operation (75). RGS is a radionuclide-based radiomic model to measure intra-operative γ-emission using ^99m^Tc- or ^111^In-labeled PSMA-ligand and provide acoustic feedback with a gamma probe (31). Schottelius et al. first reported the possibility of ^111^In-PSMA-I&T as an RGS radionuclide probe in 2015 (76). After that, Rauscher et al. conducted a comparative study in which the sensitivity, specificity, and accuracy of ^111^In-labeled PSMA RGS were 92.3%, 93.5%, and 93.1% respectively (77). ^111^In-PSMA-I&T-RGS facilitated intra-operative resection of sub-centimeter metastatic lymph nodes. The smallest resected metastatic lesion was 2 mm in size (75). In contrast, the ^99m^Tc, known as the most widely applied and available radioisotope, was introduced as a PSMA-targeted RGS probe due to its medium-energy γ-radiation, low cost, short half-life, and high stability *in vivo* in 2016 (78). In the past few years, ^99m^Tc-labeled PSMA-I&S gradually replaced the application of ^111^In-labeled PSMA-I&T in RGS (79, 80). In a retrospective analysis of 132 surgical specimens in 31 patients with BCR after primary RP who underwent ^99m^Tc-PSMA-RGS, Maurer et al. reported that this novel approach had a sensitivity of 83.6% (95% CI 70.9%, 91.5%), a specificity of 100%, and an accuracy of 93.0% (95% CI 85.8%, 96.7%) (74).

Moreover, the robot-assisted PSMA-ligand surgery has gradually become a hot spot in RGS recently. Yılmaz et al. conducted ^99m^Tc-PSMA-targeted robot-assisted RGS in 15 intermediate- or high-risk score (D’Amico risk stratification) patients; the sensitivity, specificity, accuracy, and negative and positive predictive value were 100%, respectively (81). Thus, patients with PCa profit from RGS because suspicious metastatic lymph nodes are accurately resected, which could correlate with better prognosis.

This technique has been used in PSMA-positive PCa patients; however, the correlation between PSMA IHC and PSMA uptake using gamma probe during surgery is still unclear. The activity gathered by the gamma probe during surgery is dependent not only on the specific PSMA-ligand accumulation in cells, but also on the distance of the probe to the lesions and the size of the tumor deposit. Therefore, clear correlations between the signal from the gamma probe and the PSMA expression on preclinical and clinical experiments are difficult to achieve and have not been reported so far in literature. Thus, there are no guidelines and threshold values of gamma probe counts for metastatic lesion detection.

## Conclusions

PSMA-targeted imaging and therapy represent a rapidly emerging strategy in PCa management. PSMA-ligand imaging provides accurate staging of primary PCa and high diagnostic efficacy in recurrent lesions compared with conventional imaging, allowing early therapeutic intervention. Moreover, there is evidence that the results may contribute to a change in the risk classification, and impact clinical practice. Standardized interpretation (e.g,. miTNM) of PSMA-ligand PET and its potential for predicting prognosis are evolving. Several institutions have adopted these in clinical practice. Furthermore, PSMA-targeted radioligand therapy is a very promising approach for the treatment of advanced PCa, especially mCRPC, with recent evidence showing a survival benefit. Several prospective trials are ongoing and will hopefully provide evidence for supporting approval for marketing. We believe that PSMA-targeted theranostics will soon become significant strategies in the standard of care for PCa management. Nuclear medicine physicians should be integrated into multidisciplinary teams, along with medical oncologists, radiation oncologists, urologists, and radiologists, to optimize patient care.

## Author contributions

All authors contributed to the study conception and design. Material preparation and data collection were performed by HW, GL, JZ, and ME. The first draft of the manuscript was written by HW and GL. The manuscript was reviewed by RT, and all authors commented on previous versions of the manuscript. All authors read and approved the final manuscript.
